# Prevalence of molecular markers of *Plasmodium falciparum* drug resistance in Dakar, Senegal

**DOI:** 10.1186/1475-2875-11-197

**Published:** 2012-06-13

**Authors:** Nathalie Wurtz, Bécaye Fall, Aurélie Pascual, Silmane Diawara, Kowry Sow, Eric Baret, Bakary Diatta, Khadidiatou B Fall, Pape S Mbaye, Fatou Fall, Yaya Diémé, Christophe Rogier, Raymond Bercion, Sébastien Briolant, Boubacar Wade, Bruno Pradines

**Affiliations:** 1 Unité de parasitologie - Unité de recherche sur les maladies infectieuses et transmissibles émergentes - UMR 6236, Institut de recherche biomédicale des armées, Marseille, France; 2 Laboratoire d’étude de la chimiosensibilité du paludisme, Fédération des laboratoires, Hôpital Principal de Dakar, Dakar, Sénégal; 3 Service de réanimation médicale, Hôpital Principal de Dakar, Dakar, Sénégal; 4 Service de pathologie infectieuse, Hôpital Principal de Dakar, Dakar, Sénégal; 5 Département de médecine interne et spécialités médicales de pathologie tropicale, Hôpital Principal de Dakar, Dakar, Sénégal; 6 Service interne d’hépato-gastroentérologie, Hôpital Principal de Dakar, Dakar, Sénégal; 7 Chefferie, Hôpital Principal de Dakar, Dakar, Sénégal; 8 Centre National de référence du Paludisme, Marseille, France

**Keywords:** Malaria, *Plasmodium falciparum*, Anti-malarial, *In vitro*, Resistance, Molecular marker, Senegal

## Abstract

**Background:**

As a result of the widespread resistance to chloroquine and sulphadoxine-pyrimethamine, artemisinin-based combination therapy (ACT) (including artemether-lumefantrine and artesunate-amodiaquine) has been recommended as a first-line anti-malarial regimen in Senegal since 2006. Intermittent preventive treatments with anti-malarial drugs based on sulphadoxine-pyrimethamine are also given to children or pregnant women once per month during the transmission season. Since 2006, there have been very few reports on the susceptibility of *Plasmodium falciparum* to anti-malarial drugs. To estimate the prevalence of resistance to several anti-malarial drugs since the introduction of the widespread use of ACT, the presence of molecular markers associated with resistance to chloroquine and sulphadoxine-pyrimethamine was assessed in local isolates at the military hospital of Dakar.

**Methods:**

The prevalence of genetic polymorphisms in genes associated with anti-malarial drug resistance, i.e., *Pfcrt*, *Pfdhfr*, *Pfdhps* and *Pfmdr1*, and the copy number of *Pfmdr1* were evaluated for a panel of 174 isolates collected from patients recruited at the military hospital of Dakar from 14 October 2009 to 19 January 2010.

**Results:**

The *Pfcrt* 76T mutation was identified in 37.2% of the samples. The *Pfmdr1* 86Y and 184F mutations were found in 16.6% and 67.6% of the tested samples, respectively. Twenty-eight of the 29 isolates with the 86Y mutation were also mutated at codon 184. Only one isolate (0.6%) had two copies of *Pfmdr1*. The *Pfdhfr* 108N/T, 51I and 59R mutations were identified in 82.4%, 83.5% and 74.1% of the samples, respectively. The double mutant (108N and 51I) was detected in 83.5% of the isolates, and the triple mutant (108N, 51I and 59R) was detected in 75.3%. The *Pfdhps* 437G, 436F/A and 613S mutations were found in 40.2%, 35.1% and 1.8% of the samples, respectively. There was no double mutant (437G and 540E) or no quintuple mutant (*Pfdhfr* 108N, 51I and 59R and *Pfdhps* 437G and 540E). The prevalence of the quadruple mutant (*Pfdhfr* 108N, 51I and 59R and *Pfdhps* 437G) was 36.5%.

**Conclusions:**

Since 2004, the prevalence of chloroquine resistance had decreased. The prevalence of isolates with high-level pyrimethamine resistance is 83.5%. The prevalence of isolates resistant to sulphadoxine is 40.2%. However, no quintuple mutant (*Pfdhfr* 108N, 51I and 59R and *Pfdhps* 437G and 540E), which is associated with a high level of sulphadoxine-pyrimethamine resistance, has been identified to date. The resistance to amodiaquine remains moderate.

## Background

During the past 20 years, many strains of *Plasmodium falciparum* have become resistant to chloroquine and other anti-malarial drugs [1]. One strategy for reducing malaria prevalence is the use of drugs in combination. Drug combinations help prevent the development of resistance to each component drug and reduce the overall transmission of malaria [2]. In response to increasing chloroquine resistance, Senegal in 2004 switched to sulphadoxine-pyrimethamine with amodiaquine as the first-line therapy. In 2006, artemether-lumefantrine and artesunate-amodiaquine were the forms of artemisinin-based combination therapy (ACT) recommended by the WHO as the first-line anti-malarial regimen for managing uncomplicated malaria. Since 2006, more than 1.5 million treatments have been administered in Senegal [3]. During 2009, 184,170 doses of ACT were dispensed in Senegal [4].

Dakar, the capital city of Senegal, has an urban population of approximately 1.1 million and a suburban population of 2.3 million; the city covers the majority of the Cap-Vert Peninsula. Malaria is transmitted in Dakar and its surrounding suburbs, with spatial heterogeneity of the human biting rate, which ranged from 0.1 to 250 bites per person per night during the rainy season from 2007 to 2010 [5]. Intermittent preventive treatment (IPT) with anti-malarial drugs given to all children and pregnant women once per month during the transmission season can provide a high degree of protection against malaria. Seasonal IPT with sulphadoxine-pyrimethamine and one dose of artesunate resulted in a 90% reduction in the incidence of clinical malaria in Senegal [6]. The combination of sulphadoxine-pyrimethamine and amodiaquine was more effective than the combination of sulphadoxine-pyrimethamine and artesunate or the combination of amodiaquine and artesunate in preventing malaria [7]. During IPT with sulphadoxine-pyrimethamine and piperaquine, only 3.4% of the treated children developed malaria [8].

Since the introduction of ACT and IPT trials in Senegal, there have been very few reports on the level of resistance of *P. falciparum* to anti-malarial drugs. To determine whether parasite susceptibility has been affected by the new anti-malarial policies, a study of molecular markers was conducted with local isolates obtained from the military hospital of Dakar (Hôpital Principal de Dakar). The prevalence of genetic polymorphisms in genes associated with anti-malarial drug resistance was evaluated. The genes interest included *P. falciparum* chloroquine resistance transporter (*Pfcrt*) for chloroquine [9], *P. falciparum* dihydrofolate reductase (*Pfdhfr*) for pyrimethamine [10], *P. falciparum* dihydropteroate synthase (*Pfdhps*) for sulphadoxine [11] and *P. falciparum* multidrug resistance 1 (*Pfmdr1*) for mefloquine resistance [12] and potentially for quinoline resistance [13,14].

The *Pfcrt* gene was firstly identified in 2000 [9]. So far at least 20 mutation points were described [9,15,16], but only one is the reference mutation, the marker of chloroquine resistant phenotype: K76 that becomes T76 when mutated. This mutation is often associated with other mutations in the *Pfcrt* gene (Cys72Ser, Met74Ile, Asn75Glu, Ala220Ser, Gln271Glu, Asn326Ser, Ile356Thr, Arg371Ile). The role of these mutations is not yet defined. The odds ratio (OR) for failure associated with K76T mutation was 2.1 (95% confidence interval: 1.5-3.0, meta-analysis of 13 studies) for a 14-day follow-up and 7.2 (95%CI: 4.5-11.5, meta-analysis of 12 studies) for a 28-day follow-up [17]. However, the existence of chloroquine-susceptible strains associated with K76T mutation suggests that other genes could be involved in resistance to chloroquine.

The Ser108Asn mutation on the *Pfdhfr* gene is associated with resistance to anti-folate drugs [18]. The OR for sulphadoxine-pyrimethamine failure associated with Ser108Asn was 3.5 (95%CI: 1.9-6.3, meta-analysis of 10 studies) for a 28-day follow-up [17]. The additional mutations Asn51Ile, Cys59Arg or Ile164Leu increase the level of in vitro resistance to antifolate drugs and sulphadoxine-pyrimethamine. The OR for codon 51 and 59 single mutants were 1.7 (95%CI: 1.0-3.0) and 1.9 (95%CI: 1.4-2.6), respectively [17]. The triple mutation (51+59+108) increases the risk of *in vivo* resistance to sulphadoxine-pyrimethamine by 4.3 (95%CI: 3.0-6.3, meta-analysis of 22 28-day studies) [17].

Sulphones (dapsone) and sulphonamides (sulphadoxine) are inhibitors of *P. falciparum* DHPS [19]. The mutations Ser436Ala, Ser436Phe, Ala437Gly and Lys540Glu are involved in resistance to sulphadoxine [11]. The single mutation Ala437Gly and the double mutation Ala437Gly+Lys540Glu increase the risk of *in vivo* resistance to sulphadoxine-pyrimethamine by 1.5 (95%CI: 1.0-2.4, meta-analysis of 12 studies) and 3.9 (95%CI: 2.6-5.8, meta-analysis of 10 studies), respectively [17].

The quintuple mutant of *Pfdhfr* (codons 51+59+108) plus *Pfdhps* (codons 437+540) increases the risk of *in vivo* resistance to sulphadoxine-pyrimethamine by 5.2 (95%CI: 3.2-8.8, meta-analysis of 3 studies) [17].

*Pfmdr1*, which encodes a 162 kDa protein named *P. falciparum* homologue of the P-glycoprotein (Pgh1), is located on chromosome 5. Field work has shown that the predictive value for chloroquine resistance and point mutations in the *Pfmdr1* sequence resulting in amino acid changes varies depending on the geographic area [20,21]. Five point mutations have been described: N86Y, Y184F, S1034C, N1042D and D1246Y. Point mutations, most notably N86Y, have been associated with a decrease in the chloroquine susceptibility [22]. However, in some of these epidemiological studies, the number of chloroquine-susceptible samples is too limited to provide statistically meaningful analysis [21,23]. Using precautions, no or only weak relationships are established in *P. falciparum* between chloroquine resistance and mutations in * Pfmdr1 *[24]. However, the risk of therapeutic failure with chloroquine is greater for patients harbouring the N86Y mutation with an OR of 2.2 (95%CI: 1.6-3.1) with a 14-day follow-up and 1.8 (95%CI: 1.3-2.4) with a 28-day follow-up [17]. The combination of *Pfmdr1* N86Y and *Pfcrt* K76T increases the risk of *in vivo* resistance to chloroquine by 3.9 (95%CI: 2.6-5.8, meta-analysis of 5 studies) [17].

In addition, the risk of therapeutic failure with amodiaquine is greater for patients harbouring the N86Y mutation with an OR of 5.4 (95%CI: 2.6-11.2, meta-analysis of six studies) [17]. This mutation increases the risk of failure with amodiaquine plus sulphadoxine-pyrimethamine by 7.9 [25].

It has been shown through heterologous expression that *Pfmdr1* mutations at codons 1034 and 1042 abolish or reduce the level of resistance to mefloquine [26]. Moreover, transfections with a wild-type *Pfmdr1* allele at codons 1034, 1042 and 1246 confer mefloquine resistance to susceptible parasites [27]. However, mutations at codons 1034, 1042 and 1246 in *P. falciparum Pfmdr1* isolates are not sufficient to explain variations in mefloquine susceptibility [28]. Analyses of *P. falciparum* isolates showed an association between mutation at the codon 86 and an increase in susceptibility to mefloquine, halofantrine or artemisinin derivatives [29-31].

Amplification and overexpression of *Pfmdr1* has been associated with mefloquine resistance and halofantrine decreased susceptibility in * P. falciparum *[32,33] Recently, Price *et al.* showed that amplification of *Pfmdr1* is the main cause of resistance to mefloquine in *P. falciparum*[12]; the *Pfmdr1* copy number could be used as a molecular marker to monitor mefloquine drug resistance in areas of emerging resistance [29]. The OR for mefloquine failure in monotherapy associated with *Pfmdr1* amplification is 8.6 (95%CI: 3.3-22.9) at day 28 [17]. Increased copy number from 1 to 2 is associated with a significant high risk of clinical failures with mefloquine-artesunate (OR = 2.6) [17,34] and artemether-lumefantrine [17]. Increase of *Pfmdr1* copy is associated with *in vitro* reduced susceptibility to artemisinin derivatives [35-37]. However, increase of *Pfmdr1* copy seems to be not associated with *in vivo* prolonged clearance time [38,39].

## Methods

### *Plasmodium falciparum* isolates

In total, 188 patients (109 males and 79 females) with malaria were recruited from 14 October 2009 to 19 January 2010 at the Hôpital Principal de Dakar. Venous blood samples were collected in Vacutainer® ACD tubes (Becton Dickinson, Rutherford, NJ, USA) prior to patient treatment. Parasitaemia ranged from 0.3% to 35% in male group and from 0.01% to 10% in female group. Informed verbal consent was obtained from patients and/or their parents before blood collection. The study was reviewed and approved by the ethical committee of the Hôpital Principal de Dakar. Patients were treated by artemether-lumefantrine, quinine or quinine-doxycycline.

### Nucleic acid extraction

Total genomic DNA of each strain was isolated using the QIAamp® DNA Mini kit according to the manufacturer’s recommendations (Qiagen, Germany).

### *Pfcrt* single-nucleotide polymorphisms (SNPs)

A 546-nucleotide fragment of the *Pfcrt* gene (containing codon 76) was amplified by PCR using CRTP1-sense 5’-CCG TTA ATA ATA AAT ACA CGC AG-3’ and CRTP1-antisense 5’-CGG ATG TTA CAA AAC TAT AGT TAC C-3’ primers [40]. The reaction mixture for PCR amplifications included 2.5 μl of genomic DNA, 2.5 μl of 10X reaction buffer (Eurogentec), 0.5 μM of each primer, 200 μM of a deoxynucleoside triphosphate mixture (dGTP, dATP, dTTP and dCTP) (Euromedex, Souffelweyersheim, France), 2.5 mM MgCl_2_ and 1 unit of RedGoldStar® DNA polymerase (Eurogentec) in a final volume of 25 μl. The thermal cycler (T3 Biometra, Archamps, France) was programmed as follows: an initial 94°C incubation for 5 min; 40 cycles of 94°C for 20 sec, 56°C for 20 sec and 60°C for 40 sec; and a final 5-min extension step at 60°C. The PCR products were loaded on a 1.5% agarose gel containing 0.5 μg/mL ethidium bromide. The PCR products were diluted 1:100 in distilled water, and 2.5 μl of the final dilution was used for the second PCR. This PCR amplified a 275 bp segment around the mutation using a common inner primer CRTP3-sense 5’-TGA CGA GCG TTA TAG AG-3’ coupled with either CRTP4m-antisense 5’-GTT CTT TTA GCA AAA ATT G-3’ (detects the 76 T codon) or CRTP4w-antisense 5’-GTT CTT TTA GCA AAA ATT T-3’ (detects the 76 K codon) [15]. The reaction mixture for the PCR amplifications included 2.5 μl of diluted PCR product, 2.5 μl of 10X reaction buffer (Eurogentec), 0.5 μM of each primer, 200 μM deoxynucleoside triphosphate mixture (dGTP, dATP, dTTP and dCTP) (Euromedex, Souffelweyersheim, France), 1.5 mM MgCl_2_ and 0.75 U of RedGoldStar® DNA polymerase (Eurogentec) in a final volume of 25 μl.

The PCR conditions were at 94°C for 5 min; 15 cycles at 94°C for 20 sec, 48.5°C for 20 sec and 64°C for 40 sec; and a final 5 min extension step at 64°C. Purified genomic DNA from *P. falciparum* clones 3D7 (chloroquine sensitive) and W2 (chloroquine resistant) were used as positive controls, and water and human DNA were used as negative controls. The PCR products from the amplification reactions were evaluated by electrophoresis on 2% agarose gels.

### *Pfmdr1* SNPs

Two primer pairs were used to amplify *pfmdr1* fragments carrying the five key codons [41]. A 590-base pair fragment was amplified with the primer pair sense 5’-AGA GAA AAA AGA TGG TAA CCT CAG-3’ and antisense 5’-ACC ACA AAC ATA AAT TAA CGG-3’ to determine the sequences of codons 86 and 184 (MDR1-1), and a second fragment (968 base pairs) was amplified with the primer pair sense 5’-CAG GAA GCA TTT TAT AAT ATG CAT-3’ and antisense 5'-CGT TTA ACA TCT TCC AAT GTT GCA-3' to determine the sequences of codons 1034, 1042, and 1246 (MDR1-2) [41]. The reaction mixture consisted of approximately 2.5 μl of genomic DNA, 0.5 μM of forward and reverse primers, 2.5 μl of 10X reaction buffer (Eurogentec), 2.5 mM MgCl_2_, 200 μM deoxynucleoside triphosphate mixture (dGTP, dATP, dTTP and dCTP) (Euromedex, Souffelweyersheim, France) and 1 U of RedGoldStar® DNA polymerase (Eurogentec) in a final volume of 25 μl. The thermal cycler (T3 Biometra) was programmed as follows: for MDR1-1, an initial 94°C for 5 min; 40 cycles of 94°C for 30 sec, 52°C for 30 sec and 72°C for 1 min; and a final 10-min extension step at 72°C; for MDR1-2, an initial 94°C for 5 min; 40 cycles of 94°C for 30 sec, 56°C for 1 min and 72°C for 1 min 30 sec; and a final 10-min extension step at 72°C The PCR products were loaded on a 1.5% agarose gel containing 0.5 μg/mL ethidium bromide. Amplicons were purified using the QIAquick 96 PCR BioRobot Kit and an automated protocol on the BioRobot 8000 workstation (Qiagen, Courtaboeuf, France). The purified fragments were sequenced using the BigDye Terminator v3.1 Cycle Sequencing Kit (Applied Biosystems) using the primers described above. The sequencing reaction products were purified using the BigDye XTerminator® Purification Kit (Applied Biosystems) in accordance with the manufacturer's instructions. The purified products were sequenced using an ABI Prism 3100 analyser (Applied Biosystems). Sequences were analysed using Vector NTI advance(TM) software (version 11, Invitrogen, Cergy Pontoise, France).

### ***Pfdhfr*SN**P

A 562-bp fragment corresponding to the coding region of *Pfdhfr* was amplified using the following primers: sense 5’-ACG TTT TCG ATA TTT ATG C-3’ and antisense 5’ TCA CAT TCA TAT GTA CTA TTT ATT C-3’ [42]. The reaction mixture contained 2.5 μl of genomic DNA, 2.5 μl of 10X reaction buffer (Eurogentec), 0.5 μM each primer, 2.5 mM MgCl_2_, 200 μM deoxynucleoside triphosphate mixture (dGTP, dATP, dTTP and dCTP) (Euromedex, Souffelweyersheim, France) and 1 U of RedGoldStar® DNA polymerase (Eurogentec) in a final volume of 25 μl. The PCR conditions were as described in [42]. The amplified fragments were purified, sequenced (with the primers used for PCR) and analysed as described above.

### *Pfdhps* SNP

A 672-bp fragment corresponding to the coding region of *Pfdhps* was amplified using the following primers: sense 5′-GTT GAA CCT AAA CGT GCT GT-3′ and antisense 5′-TTC ATC ATG TAA TTT TTG TTG TG-3′ [42]. The fragment was amplified as described for *pfdhfr*, and the PCR conditions were as described in [42]. The amplified fragments were purified, sequenced (with the primers used for PCR) and analysed as described above.

### Copy number of *Pfmdr1*

The *Pfmdr1* copy number was estimated by TaqMan real-time PCR (7900HT Fast Real-Time PCR system, Applied Biosystems, Courtaboeuf, France) using the single-copy gene *β-tubulin* (PF10_0084) as a reference. The following previously reported oligonucleotide primers and probes were used with slight modifications [12]: 5’-TGC ATC TAT AAA ACG ATC AGA CAA A-3’, 5’-TCG TGT GTT CCA TGT GAC TGT-3’ and 5’-VIC- TTT AAT AAC CCT GAT CGA AAT GGA ACC TTT G-TAMRA-3’ for *Pfmdr1* and 5’- TGA TGT GCG CAA GTG ATC C-3’, 5’-TCC TTT GTG GAC ATT CTT CCT C-3’ and 5’-FAM- TAG CAC ATG CCG TTA AAT ATC TTC CAT GTC T-TAMRA-3’ for *β-tubulin* (Eurogentec, Angers, France). Individual PCR reactions were carried out using 1X TaqMan Universal PCR Master Mix (Applied Biosystems), 900 nM forward primer, 900 nM reverse primer, 250 nM Taqman probe and 5 μl of template DNA in a final volume of 25 μl. The reaction mixtures were prepared at 4°C in a 96-well optical reaction plate (Applied Biosystems) covered with optical adhesive covers (Applied Biosystems). The thermal cycling conditions were 50°C for 2 min, 95°C for 10 min, and 50 cycles of 95°C for 15 s and 60°C for 1 min. Each sample was assayed in triplicate, and the data were analysed with SDS software 2.2.1 (Applied Biosystems). The PCR efficiencies of all primer pairs were evaluated using a dilution series of *P. falciparum* 3D7 genomic DNA and were found to be sufficiently close to obviate the need for any correction factor. Therefore, the 2^-ΔΔCt^ method of relative quantification was used and adapted to estimate the copy number of the *pfmdr1* gene, where ΔΔCt = (Ct_*pfmdr1*_ - Ct_*β-tubuline*_)_sample_ - (Ct_*pfmdr1*_ - Ct_*β-tubuline*_)_calibrator_. Genomic DNA extracted from the *P. falciparum* 3D7 strain, which has a single copy of each gene, was used as a calibrator, and *β-tubulin* served as the control housekeeping gene in all experiments.

## Results

One hundred and eighty-eight patients were recruited for the study at the Hôpital Principal de Dakar. Gene polymorphisms were evaluated in 174 isolates that were slide-positive for *P. falciparum*.

*Pfcrt* was examined in 164 of the *P. falciparum*-positive samples. At the *Pcrt* gene, the codon 76 (K76T) mutation was identified in 37.2% of the samples. One sample (0.6%) was mixed, yielding both K76 and 76T.

The results for *Pfmdr1* polymorphisms are shown in Table 1. The codon 86 (N86Y) mutation was identified in 16.6% of the tested samples. One isolate (0.6%) was mixed, yielding both N86 and 86Y. A mutation in codon 184 (Y184F) was identified in 67.6% of the isolates. Twenty-eight of the 29 isolates with the 86Y mutation were also mutated at codon 184 (184F). No new SNP was detected in *Pfmdr1* gene. Only one isolate (0.6%) harboured two copies of *pfmdr1*.

**Table 1:** **Number (no) and frequency (%) of the* Pfmdr1 *mutations (codons 86, 184, 1034, 1042, and 1246)**

**Codon**	**No**	**Wild type no (%)**	**Mutated no (%)**	**Wild type/ Mutated no (%)**
N86Y	174	144 (82.8)	29 (16.6)	1 (0.6)
Y184F	173	56 (32.4)	117 (67.6)	0 (0)
S1034C	163	163 (100)	0 (0)	0 (0)
N1042D	170	170 (100)	0 (0)	0 (0)
D1246Y	168	168 (100)	0 (0)	0 (0)

The results for *Pfdhfr* polymorphisms are presented in Table 2. There was a mutation in 82.4% of the samples for codon 108 (S108N/T) (only 1/140 S108N/T was S108T), in 83.5% for codon 51 (N51I) and in 74.1% for codon 59 (C59R). Nine samples (5.2%), one sample (0.6%) and 19 samples (11.2%) were mixed, yielding both S108 and 108N, N51 and 51I, and C59 and 59R, respectively. The double mutant (108N and 51I) was detected in 83.5% of the isolates, and the triple mutant (108N, 51I and 59R) was detected in 75.3%. Two mutants were detected for codon 16 (A16V).

**Table 2:** **Number (no) and frequency (%) of the* Pdhfr *mutations (codons 108, 51, 59, 16, and 164)**

**Codon**	**No**	**Wild type no (%)**	**Mutated no (%)**	**Wild type/ Mutated no (%)**
S108N/T*	170	21 (12.4)	140* (82.4)	9 (5.2)
N51I	170	27 (15.9)	142 (83.5)	1 (0.6)
C59R	170	25 (14.7)	126 (74.1)	19 (11.2)
A16V	158	155 (98.1)	2 (1.3)	1 (0.6)
I164L	170	169 (99.4)	1 (0.6)	0 (0)

*One was 108T.

The results for *Pfdhps* polymorphisms are presented in Table 3. There was a mutation in 40.2% of the samples for codon 437 (A437G), in 35.1% for codon 436 (S436F/A) and in 1.8% for codon 613 (A613S). No mutant was detected for codons 540 and 581.

**Table 3:** **Number (no) and frequency (%) of the* Pfdhps *mutations (codons 437, 436, 540, 581, and 613)**

**Codon**	**No**	**Wild type no (%)**	**Mutated no (%)**	**Wild type/ Mutated no (%)**
A437G	174	104 (59.8)	70 (40.2)	0 (0)
S436F/A	174	112 (64.3)	61 (35.1)	1 (0.6)
K540E	174	174 (100)	0 (0)	0 (0)
A581G	174	174 (100)	0 (0)	0 (0)
A613S	171	168 (98.2)	3 (1.8)	0 (0)

There was no double mutant (437G and 540E) and no quintuple mutant (*Pfdhfr* 108N, 51I and 59R and *Pfdhps* 437G and 540E). The prevalence of the quadruple mutant (*Pfdhfr* 108N, 51I and 59R and *Pfdhps* 437G) was 36.5%.

## Discussion

In response to increasing chloroquine resistance, Senegal in 2004 switched to sulphadoxine-pyrimethamine with amodiaquine as the first-line therapy. In 2006, artemether-lumefantrine and artesunate-amodiaquine were the forms of artemisinin-based combination therapy (ACT) recommended by the WHO as the first-line anti-malarial regimen for managing uncomplicated malaria.

Mutations in *Pfcrt* have been shown to be correlated with chloroquine resistance in different parts of the world [43]. The prevalence of the *Pfcrt* 76T mutation decreased since 2004 in Dakar. In 2000–2001 in Guediawaye, a suburb of Dakar, a prevalence of 92% of 76T was observed in pregnant women with malaria [44]. In Pikine, another suburb of Dakar, the prevalence of 76T was 79% in 2000 [45], 63.9% in 2001 [46] and 59.5% in 2004 [47]. In 2002, the prevalences of *in vitro* resistance to chloroquine and of *Pfcrt* 76T mutation were 52% and 65%, respectively, in patients hospitalized for malaria at the Hôpital Principal de Dakar [48]. In 2001–2002, the prevalence of the *pfcrt* 76T mutation was 75.8% in pregnant women taking chloroquine prophylaxis in Thiadiaye (84 km southeast of Dakar) [49]. In Dielmo (280 km southeast of Dakar), the *in vitro* resistance to chloroquine regularly increased from 32% in 1995 to 55% in 1999 [50-53].

In this study, the *Pfcrt* 76T mutation was identified in 37.2% of the patients recruited from October 2009 to January 2010 in the Hôpital Principal de Dakar. These data are consistent with previous works on molecular resistance and on *in vitro* or *ex vivo* susceptibility in Dakar in 2009 (22% of isolates exhibiting chloroquine resistance) [54] and in Thies in 2007 (23% of isolates exhibiting chloroquine resistance) [55].

This decrease in chloroquine resistance parallels the withdrawal of chloroquine treatment and the introduction of ACT in 2002 in Senegal. However, in 2003, chloroquine was still being administered to patients. The prevalence of chloroquine in the urine ranged from 14.5% to 47.5% in two- to nine-year-old children from northern Senegal and from 9.0% to 21.4% in children from southern Senegal [56]. In 2006, Senegal reported 10.6% chloroquine use and 9.7% ACT use [57]. Since 2006, more than 1.5 million ACT treatments have been administered in Senegal [3], and 184,170 doses of ACT were dispensed in 2009 [4]. A reduction in chloroquine resistance was also reported in Malawi after the withdrawal of chloroquine treatment [58]. This observation prompted an *in vivo* chloroquine study in Malawi five years later, in which chloroquine was found to be 99% effective [59]. The rapid dissemination of chloroquine resistance in Dielmo, despite strictly controlled anti-malarial drug use, argues against the re-introduction of chloroquine at least in mono-therapy in places where the resistant allele has dropped to very low levels following the discontinuation of chloroquine treatment [60]. It took 407 chloroquine treatments (1.6 treatment courses/person/year) in the community to raise the prevalence of the *Pfcrt* 76T mutation from an 8-9% during the first year of re-introduction of chloroquine (1993–1994) to 46% in 1995. Increased selective pressure (2752 treatments during the period 1995–1999) did not increase the prevalence of *Pfcrt* 76T or increased the *in vitro* resistance to chloroquine, but this increased selective pressure increased the incidence of clinical malaria for patients within seven days of chloroquine treatment from 2.6% in 1995 to 13% in 1999.

The *Pfdhfr* 108N mutation has been shown to be correlated with *in vitro* and *in vivo* resistance to pyrimethamine [10,17]. The OR for sulphadoxine-pyrimethamine failure associated with Ser108Asn was 3.5 (95%CI: 1.9-6.3, meta-analysis of 10 studies) for a 28-day follow-up [17]. The additional mutations Asn51Ile, Cys59Arg or Ile164Leu increase the level of in vitro resistance to anti-folate drugs and sulphadoxine-pyrimethamine. The OR for codon 51 and 59 single mutants were 1.7 (95%CI: 1.0-3.0) and 1.9 (95%CI: 1.4-2.6), respectively [17]. In 2009, the prevalence of *Pfdhfr* 108N was 82.4% in patients with malaria who were treated at the Hôpital Principal de Dakar. The triple mutation (51+59+108) increases the risk of *in vivo* resistance to sulphadoxine-pyrimethamine by 4.3 (95%CI: 3.0-6.3, meta-analysis of 22 28-day studies). Isolates carrying a combination of three mutations (108N, 51I and 59R) associated with high-level pyrimethamine resistance represented 75.3%. In 2002, in the same hospital, the prevalence of *Pfdhfr* 108N was 65%, and triple mutants were identified in 50% of the isolates [48]. In 2003, the prevalence of mutations in *Pfdhfr* codon 108 was 78% in Pikine, and the prevalence of the triple mutant was 61% [61]. In 2007 in Keur Soce, a rural area, triple mutant was identified in 67% of patients treated with sulphadoxine-pyrimethamine combined with amodiaquine [8].

The *Pfdhps* 437G mutation has been shown to be correlated with *in vitro* and *in vivo* resistance to sulphadoxine [11,17]. The single mutation Ala437Gly and the double mutation Ala437Gly+Lys540Glu increase the risk of *in vivo* resistance to sulphadoxine-pyrimethamine by 1.5 (95%CI: 1.0-2.4, meta-analysis of 12 studies) and 3.9 (95%CI: 2.6-5.8, meta-analysis of 10 studies), respectively [17]. In 2009, the prevalence of the *Pfdhps* 437G mutation was 40.4% in patients with malaria who were treated at the Hôpital Principal de Dakar. However, there was no isolate carrying the double mutation (437G and 540E) that is associated with high-level sulphadoxine resistance. The mutation of codon 613 (A613S) (1.8%) was very rare in Africa. In 2002, in the same hospital, only 20% of isolates harboured the *Pfdhps* 437G mutation [48]. In 2003, the mutation rate in *Pfdhps* codon 437G was 40% in Pikine [61]. Several studies from 2006 to 2008 in Senegal showed that the prevalence of *Pfdhps* 437G significantly increased after intermittent preventive treatment of infants with sulphadoxine-pyrimethamine [8,62]. Given the prevalences of the triple and quadruple mutants in the population of Dakar (75.3% *Pfdhfr* 108N, 51I and 59R triple mutant and 36.5% quadruple mutant *Pfdhfr* 108N, 51I and 59R and *Pfdhps* 437G), the use of sulphadoxine-pyrimethamine as an intermittent preventive treatment must be monitored. Encouragingly, no quintuple mutant (*Pfdhfr* 108N, 51I and 59R and *Pfdhps* 437G and 540E), which is associated with high-level sulphadoxine-pyrimethamine resistance, has been identified to date. However, the single use of sulphadoxine-pyrimethamine as seasonal IPT must be inadvisable: sulphadoxine-pyrimethamine must be associated with amodiaquine, artesunate or piperaquine for instance [7,8].

Only one isolate (0.6%) had two copies of *Pfmdr1*. In Asia, amplification of *Pfmdr1* is associated with mefloquine resistance *in vitro* and *in vivo*[12,29,63]. The role of increased copy number in mefloquine resistance in Africa remains controversial. Isolates with a duplicated *Pfmdr1* gene circulate in West Africa but are relatively rare [64]. Only one published clinical failure for mefloquine in West Africa was found to be associated with *in vitro* resistance and amplification of * Pfmdr1 *[65]. In addition, amodiaquine resistance is not related to the amplification of * Pfmdr1 *[66]. The role of the amplification of *Pfmdr1* in resistance to artemether-lumefantrine in Africa is still debated. It seems that no *Pfmdr1* gene amplification was associated with artemether-lumefantrine failures in Africa [67], whereas a copy number ≥4 is associated with reduced *in vitro* susceptibility to lumefantrine [68]. In 2009, in Dakar, only 1% of the isolates presented reduced *in vitro* susceptibility to lumefantrine [44], and this prevalence did not increase in Senegal after the introduction of ACT. In 1996, 6% of isolates from Dielmo were resistant *in vitro* to lumefantrine [69]. In recent years, the efficacy of artemether-lumefantrine in several trials in Senegal has ranged from 96 to 100% [70-72].

The prevalences of the *Pfmdr1* mutations 86Y and 184F were 16.6% and 67.6%, respectively. No isolate carried a mutation in codons 1034, 1042 or 1246. In 2000 and 2001, prevalences of 31% and 30.6 were observed for *Pfmdr1* 86Y in Pikine [45,46]. The role of polymorphism in *Pfmdr1* is still debated. Point mutations in *Pfmdr1*, most notably at codon 86, have been found to be associated with decreased chloroquine susceptibility [73]. Nevertheless, this association is not a consistent finding [24]. The *Pfmdr1* 86Y mutation also has been found to be associated with increased susceptibility to mefloquine or artemisinin [29-31]. This association, too, is not a consistent finding [63]. In addition, no clear association between the *Pfmdr1* 184F mutation and mefloquine failure has been established, although this allele is widespread in Cambodia [74]. The *Pfmdr1* 86Y mutation has also been shown to be associated with *in vivo* resistance to amodiaquine in recrudescence after monotherapy with amodiaquine [75] or after combination therapy with artesunate-amodiaquine [76]. The *Pfmdr1* 1246Y mutation has also been found to be associated with *in vitro* resistance to amodiaquine [77] and with recrudescent infection after treatment with amodiaquine or amodiaquine-artesunate [76,78]. In a meta-analysis, the *Pfmdr1* 86Y mutation was demonstrated to be associated with amodiaquine failure, with an odds ratio of 5.4 [17]. Based on this hypothesis, the 16.6% prevalence of *Pfmdr1* of 86Y predicts that 16.6% of isolates would be resistant to amodiaquine in 2009 in Senegal. In 2009, in Dakar, only 6% of isolates showed *in vitro* reduced susceptibility to monodesethylamodiaquine, the active metabolite of amodiaquine [54]. The resistance to amodiaquine has remained low even after the introduction of artesunate-amodiaquine in 2006 in Senegal relative to the resistance prevalences of Dielmo in 1996 and 1999 (0%) [50,79] and Mlomp in Casamance, south-western Senegal, in 2004 (5%) [80]. The artesunate-amodiaquine–associated cure rates were >99.3% in Mlomp and Keur-Socé when administered either as a single daily dose or two daily doses [81]. The fixed-dose combination of artesunate-amodiaquine (ASAQ) exhibits a cure rate >98.5% [82]. The cure rates were 100% in the populations experiencing a second or third episode of uncomplicated malaria following treatment with ASAQ [69]. However, ACT efficacy and resistance must be monitored because the first clinical failures, or at least extended parasite clearance times, have been described in Cambodia [38,83]. In this context, it is important to implement *in vitro* and *in vivo* surveillance programmes, such as those championed by the Worldwide Antimalarial Resistance Network [84,85].

Since 2004, the prevalence of chloroquine resistance has decreased, but the data argue against the re-introduction of chloroquine at least in mono-therapy in places where the resistant allele has dropped to very low levels following discontinuation of chloroquine treatment. The prevalence of isolates resistant to pyrimethamine is high (82.4%), with 75.3% of parasites exhibiting high-level pyrimethamine resistance. The prevalence of isolates resistant to sulphadoxine was 40.2%. However, no quintuple mutant (*Pfdhfr* 108N, 51I and 59R and *Pfdhps* 437G and 540E), which is associated with high-level sulphadoxine-pyrimethamine resistance, has been identified to date. The resistance to amodiaquine remains moderate. Intensive surveillance of *P. falciparum* susceptibility to anti-malarial drugs must be conducted regularly in Senegal. However, maximizing the efficacy and longevity of ACT as a tool to control malaria will critically depend on pursuing intensive research into identifying in vitro markers as well as implementing in vitro and in vivo surveillance programs. In this context, there is a need to identify molecular markers that predict ACT resistance which can provide an active surveillance method to monitor temporal trends in parasite susceptibility.

## Consent

The study was reviewed and approved by the ethics commission of Hôpital Principal de Dakar.

## Competing interests

The authors declare that they have no competing interests.

## Authors’ contributions

NW, AP, EB and SB carried out the molecular genetic studies. BF, SD, SD, TD, BD, KBF, PSM and FF carried out diagnostic tests, monitored the patients, collected clinical and epidemiological data and drafted the manuscript. CR, RB, BW and BP conceived and coordinated the study. SB, CR and BP analysed the data. NW, AP, SB and BP drafted the manuscript. All authors read and approved the final manuscript.
